# 10 Evaluation of Cefepime Pharmacokinetics in Patients with Large Burns That Received Therapeutic Drug Monitoring

**DOI:** 10.1093/jbcr/irae036.010

**Published:** 2024-04-17

**Authors:** Zachary Drabick, Albert Tieu, Ian R Driscoll, Andrea Munden, Amalia Cochran

**Affiliations:** UF Health Shands Burn Center, High Springs, Florida; Providence St. Peter, Olympia, Washington; University of Florida College of Medicine, Gainesville, FL; Department of Surgery, University of Florida College of Medicine, Gainesville, Florida; UF Health Shands Burn Center, High Springs, Florida; Providence St. Peter, Olympia, Washington; University of Florida College of Medicine, Gainesville, FL; Department of Surgery, University of Florida College of Medicine, Gainesville, Florida; UF Health Shands Burn Center, High Springs, Florida; Providence St. Peter, Olympia, Washington; University of Florida College of Medicine, Gainesville, FL; Department of Surgery, University of Florida College of Medicine, Gainesville, Florida; UF Health Shands Burn Center, High Springs, Florida; Providence St. Peter, Olympia, Washington; University of Florida College of Medicine, Gainesville, FL; Department of Surgery, University of Florida College of Medicine, Gainesville, Florida; UF Health Shands Burn Center, High Springs, Florida; Providence St. Peter, Olympia, Washington; University of Florida College of Medicine, Gainesville, FL; Department of Surgery, University of Florida College of Medicine, Gainesville, Florida

## Abstract

**Introduction:**

Therapeutic drug monitoring (TDM) can be utilized to calculate patient-specific pharmacokinetic (PK) parameters and adjust dosing. Our burn center routinely uses cefepime TDM to adjust dosing after initiation, however optimization of empiric dosing would be ideal. The objective of this study was to characterize cefepime PK parameters in burn patients as compared to a non-burn population and identify those that had significant predictive value.

**Methods:**

A retrospective chart review was conducted on patients 18 to 89 years of age admitted to a 27 bed burn center who had at least one cefepime concentration resulted between August 2019 and August 2022. Patients were excluded if only peak cefepime concentration data was available. Only index cefepime concentrations were evaluated. The primary outcome was to assess differences in pharmacokinetic values between patients with burn injuries and those without. Secondary outcomes were the incidence of estimated free minimum cefepime concentration (fCmin) ≥8 mcg/mL as the empiric therapeutic goal and fCmin ≥32 mcg/mL as the empiric prevention of resistance goal. Regression analysis was performed to identify patient characteristics significantly predictive of PK parameters.

**Results:**

Seventy-four patients were included in the final analysis (48 in the burn group and 26 in the non-burn group). In the burn cohort the median TBSA was 26.8% and a majority had a TBSA >20% (60%). Primary analysis yielded no statistically significant differences in median elimination rate constant (Ke), half-life (T1/2), or volume of distribution (Vd) between the 2 groups, though the burn cohort reached a significantly lower median Cmin as compared with the non-burn cohort (p=0.04). Linear regression analysis of the burn cohort indicated creatinine clearance (CrCl) and serum creatinine (SCr) as having significant positive correlation with Ke (p < 0.01) and T1/2 (p < 0.01) and total IV fluid volume received in the previous 24 hours as having a significant positive association with Vd (p=0.022). Secondary results showed that the burn cohort had a lower incidence of achieving estimated fCmin ≥8 mcg/mL (67% vs 88%, p=0.032) as compared to the non-burn cohort. Both groups had low attainment of fCmin ≥32 mcg/mL (19% for burn, 12% for non-burn).

**Conclusions:**

PK parameters were not statistically different between the burn and non-burn groups, though Ke and Vd were numerically larger in the burn group. CrCl, SCr were predictive of Ke and T1/2 while IV fluid volume received in the previous 24 hours was predictive of Vd. Significantly less burn patients achieved empiric cefepime concentration goals for therapeutic efficacy. Further analyses are planned to assess and optimize initial cefepime regimens to ensure better empiric target attainment.

**Applicability of Research to Practice:**

Cefepime TDM in burn patients may be useful in identifying those who are at risk of not meeting empiric goal serum concentrations so that dosing can be optimized.

